# Development and feasibility of a Swallowing intervention Package (SiP) for patients receiving radiotherapy treatment for head and neck cancer—the SiP study protocol

**DOI:** 10.1186/s40814-016-0079-6

**Published:** 2016-08-04

**Authors:** Mary Wells, Emma King, Kate Toft, Fiona MacAulay, Joanne Patterson, Nadine Dougall, Nick Hulbert-Williams, Sally Boa, Eleanor Slaven, Julie Cowie, John McGarva, Patricia Gail Niblock, Julie Philp, Justin Roe

**Affiliations:** 1 NMAHP Research Unit, University of Stirling, Stirling, UK; 2 NHS Lothian, Edinburgh, UK; 3 NHS Tayside, Dundee, UK; 4 City Hospitals Sunderland NHS Foundation Trust, Sunderland, UK; 5 NMAHP Research Unit, University of Stirling, Stirling, UK; 6 Department of Psychology, University of Chester, Chester, UK; 7 Strathcarron Hospice, Denny, UK; 8 NHS Greater Glasgow and Clyde, Glasgow, UK; 9 Faculty of Health Sciences and Sport, University of Stirling, Stirling, UK; 10 NHS Forth Valley, Larbert, UK; 11 NHS Tayside, Dundee, UK; 12 NHS Fife, Dunfermline, UK; 13 The Royal Marsden NHS Foundation Trust, London, UK

**Keywords:** Dysphagia, Cancer, Head and neck, Intervention, Exercise, Rehabilitation, Study protocol

## Abstract

**Background:**

Head and neck cancer (HNC) is the sixth most common cancer worldwide, and the functional, psychological and social consequences of HNC cancer and its treatment can be severe and chronic. Dysphagia (swallowing problems) affects up to two thirds of patients undergoing combined chemoradiotherapy. Recent reviews suggest that prophylactic swallowing exercises may improve a range of short- and long-term outcomes; however, the importance of psychological and behavioural factors on adherence to swallowing exercises has not been adequately studied. This study aims to develop and test the feasibility of a Swallowing intervention Package (SiP) designed in partnership with patients, speech and language therapists (SLTs) and other members of the head and neck multi-disciplinary team (MDT), for patients undergoing chemoradiotherapy (CRT) or radiotherapy (RT) for head and neck cancer.

**Methods/design:**

This feasibility study uses quantitative and qualitative research methods, within a quasi-experimental design, to assess whether patients will tolerate and adhere to the SiP intervention, which aspects of the intervention can be implemented and which cannot, whether treatment fidelity can be achieved across different contexts, whether study processes and outcome measures will be feasible and acceptable and to what extent the intervention is likely to have an impact on swallowing dysfunction and quality of life. Patients are being recruited from five sites in Scotland and England (three interventions and two usual care). The SLT based in the relevant intervention centre teaches the exercise programme and provides supporting materials. A combination of patient-reported outcome measures (PROMs), adherence measures and clinical swallowing assessments are used prior to intervention (baseline), at the end of treatment, 3 and 6 months post-treatment.

**Discussion:**

This collaborative study has taken a unique approach to the development of a patient-centred and evidence-based swallowing intervention. The introduction of an e-SiP app provides an exploration of the use of technology in delivering this intervention. The study provides an opportunity to examine the feasibility of delivering and participating in a supported swallowing intervention across several different NHS sites and will provide the evidence needed to refine intervention and study processes for a future trial.

**Trial registration:**

NCRI portfolio, 18192 & 20259

## Background

Head and neck cancer (HNC) is the sixth most common cancer worldwide [1]. Currently, there are more than 62,000 people living with head and neck cancer in the UK [2]. In Scotland, where incidence rates are significantly higher than the rest of the UK, a 37 % rise is predicted in the next 10 years [3]. Patients are surviving longer and, due to human papillomavirus, are younger at diagnosis. The functional, psychological and social consequences of HNC cancer and its treatment can be severe and chronic. Treatment includes a combination of surgery, radiotherapy and/or chemotherapy, and the side effects of these can interfere with some of the most fundamental aspects of daily living, including eating, drinking, communication and appearance. Improved survival has been achieved at the expense of increased morbidity, especially in relation to swallowing problems or ‘dysphagia’ [4].

Dysphagia affects up to two thirds of patients undergoing combined chemoradiotherapy (CRT) [5]. Patients report major deterioration in their swallowing, and little evidence of recovery over time, with younger survivors reporting the most severe problems [6]. Early side effects of CRT^1^ include pain, mucositis (inflammation of the mucous membranes) and xerostomia (dry mouth). Late effects include fibrosis (scarring) of the soft tissues, affecting the safety and efficiency of swallowing [7, 8]. Aspiration rates (food or fluid entering the airway) are high (≥60 %), leading to frequent hospitalisation for chest infection, pneumonia and even death [5, 8]. The effects of CRT contribute to significant weight loss, and 50–70 % of patients require a feeding tube during or after treatment [9]. Tube dependency reduces the need to swallow, so increasing the likelihood of fibrosis of the muscles involved in swallowing, and in the long-term, ‘disuse atrophy’, sometimes leading to complete inability to swallow. Swallowing difficulties have a significant negative emotional and physical impact on social eating, return to work and everyday quality of life [7].

Recent reviews [10, 11] suggest that prophylactic swallowing exercises may improve a range of short- and long-term outcomes, as they increase the blood flow to muscles, reducing or preventing fibrosis, and maintaining the range and speed of swallowing movements. However, not all trials have had positive results [12–14], and a number of questions remain, including the optimal timing, selection and duration of exercises, the achievement of intervention fidelity and, importantly, the support necessary to improve adherence [10]. Only 13–14 % of participants practise swallowing exercises as recommended [15, 16]. An unpublished study found that patients with high levels of pain during treatment struggled with performing regular exercises and that it was difficult to focus on preventative exercises in the context of facing a potentially life-limiting disease. Furthermore, the intervention was not seen as integral to their overall care [17]. There is, however, evidence that those who maintain their exercise schedule achieve improved swallowing outcomes [18] and are significantly less likely to need a feeding tube [19]—an important consideration given that feeding tubes are associated with significantly poorer quality of life [20].

The multi-dimensional nature and impact of swallowing difficulties and the importance of psychological and behavioural factors on adherence to swallowing exercises has not been adequately studied [21, 22]. The need for further research, particularly prospective randomised trials to investigate the benefit of an intervention which includes pre-, peri- and post-treatment swallowing exercises has been highlighted repeatedly [10, 23, 24].

This study aims to develop and test the feasibility of a Swallowing intervention Package (SiP) designed in partnership with patients, speech and language therapists (SLTs) and other members of the head and neck multi-disciplinary team (MDT), for patients undergoing primary or adjuvant CRT or radiotherapy for head and neck cancer. A development phase (phase 1), informed by focus groups with patients and consensus workshops with professionals, produced a swallowing exercise schedule, staff manual and SiP package for patients, including written materials, videos, reminder materials and an electronic e-SiP ‘app’ to support adherence. This protocol, based on version 5 (24.2.16) describes the feasibility study (phase 2) for this complex intervention, which aims to answer four research questions in preparation for a future multi-centre trial:

1. What is the feasibility of delivery and potential impact of the SiP for patients and head and neck cancer teams?
2. What are the barriers and facilitators to adherence and fidelity to the SiP?
3. Does an e-support system (e-SiP) have potential to support patients to perform their exercises and for collecting patient-reported data through video diaries?
4. Are the study processes and outcome measures feasible and acceptable to patients and staff?

## Methods/design

This feasibility study uses quantitative and qualitative research methods, within a quasi-experimental parallel group design, to assess whether patients will tolerate and adhere to the SiP intervention, which aspects of the intervention can be implemented and which cannot, whether treatment fidelity can be achieved across different contexts, whether study processes and outcome measures will be feasible and acceptable and to what extent the intervention is likely to have an impact on swallowing dysfunction and quality of life.

Forty patients will be recruited to the SiP intervention group, from three hospital outpatient settings in Scotland, and a maximum of 30 patients will be recruited to a ‘usual care’ group, from sites in Scotland and the North East of England. This is a pragmatic design to allow for different service delivery models in different geographical areas. There is a trade-off between precision (recruitment parameter of numbers retained by study endpoint) and number of patients recruited to the study. A sample size of 59 is thought to be sufficient to identify any potential problems in feasibility which have a 5 % probability of occurrence at least once, with 95 % confidence [25]. Several new sites have expressed an interest in participating and are being adopted in order to enhance recruitment.

To identify the variety of practical issues and problems faced by a ‘real world’ and inclusive ‘typical’ cohort of patients with head and neck cancer and the applicability of the intervention across this cohort, we will include people with/without prophylactic feeding tubes, from different demographic, diagnostic (e.g. oral, pharyngeal, laryngeal) and treatment (CRT and radiotherapy alone) groups.

Inclusion criteria:

Patients diagnosed with an index primary mucosal squamous cell carcinoma of oral cavity, nasopharynx, pharynx or larynx or unknown primary, who are planned for chemoradiotherapy or radiotherapy ≥30 fractions. This may include patients who have undergone surgery before their chemoradiation or radiotherapy. Patients must be able to give informed consent.

Exclusion criteria:

Patients with synchronous or metachronous head and neck cancer primaries, those undergoing palliative treatment, non­English speakers, previously diagnosed dysphagia unrelated to HNC and those who have undergone total glossectomy or total laryngectomy.

### Screening, recruitment and consent

All potentially eligible patients will be identified from the head and neck cancer MDT meetings. Eligible patients will be seen at routine appointments for treatment planning and invited to participate in the study by a member of the clinical team. The e-SiP app is being tested in Tayside, where patients eligible for the intervention are also eligible for the app version. The delegated individual will explain the study to the patient, give them the Patient Information Sheet (PIS) and answer any questions. Consent will be sought a minimum of 24 h later by a research nurse. The PIS for the intervention group explains that a Swallowing intervention Package of exercises and support has been developed and that we are now asking people to try this out and report their experiences, with the hope that we will be able to use these findings to underpin a larger trial. The PIS for the control group explains that patients will have no change to their usual care and will continue to have the swallowing support normally provided by their treatment centre. Both intervention and control patients are informed that taking part in the study will not affect any usual treatment that they may receive and that taking part may not benefit them personally.

At the time of consent the participant will also be asked to fill in the baseline study questionnaires, and a standard letter will be sent to their GP specifying the recruiting hospital and whether or not the patient is in the intervention or ‘usual care’ group. All patients will also be asked whether they and/or a close family member or friend would be willing to be approached for a subsequent qualitative interview with the research fellow, between 6 weeks and 3 months after treatment finishes, to explore their experiences.

In order to assess the representativeness of the sample, a screening log will record the number of patients who are (a) ineligible, (b) refuse to participate in the intervention or control cohorts and (c) decline specific aspects of the study (e.g. clinical assessments, questionnaires), as well as their reasons, where possible. The rights of patients to refuse to participate or withdraw without giving reasons will be respected. If patients do volunteer a reason for refusal, or for wishing not to use the e-SiP app versions, then these will be logged.

### Clinical and socioeconomic characteristics

At the point of recruitment, all participants are allocated a unique ID number. At baseline, the following data will be extracted from NHS patient records and recorded by research nurses/clinical teams in the study case report form:

Date of birth, sex, ethnicity, Index of Multiple Deprivation derived from patient’s residential postcode, education level, work status, social support, tumour type, anatomic site, TNM stage, size and position of primary tumour, smoking (current/ex-smoker and pack years), use of alcohol, body mass index, weight, surgery, planned CRT regime including chemotherapy regime, type of radiotherapy (e.g. intensity-modulated radiation therapy (IMRT) or conventional), number of fractions, radiation field, feeding tube and feeding regime (whether in situ or planned).

At the end of treatment, CRT details will be checked and amended as necessary to record the treatment actually delivered, including overall radiation dose to primary clinical target volume and dose to the constrictor muscles. Additional data will be recorded on:

Method of feeding throughout treatment, any admissions to hospital, analgesic regime, treatment complications and weight loss will be recorded on the post-treatment case report form and updated as appropriate. Any adverse events which are not expected as a relatively common side effect of CRT (e.g. aspiration pneumonia, death) will also be recorded on this form.

All case report forms are pseudonymised by their unique ID number, before being transferred to the Research Team for further analysis. Relevant staff within the project team have undergone Good Clinical Practice (GCP) training.

### Outcome measures

**Fig. 1:**
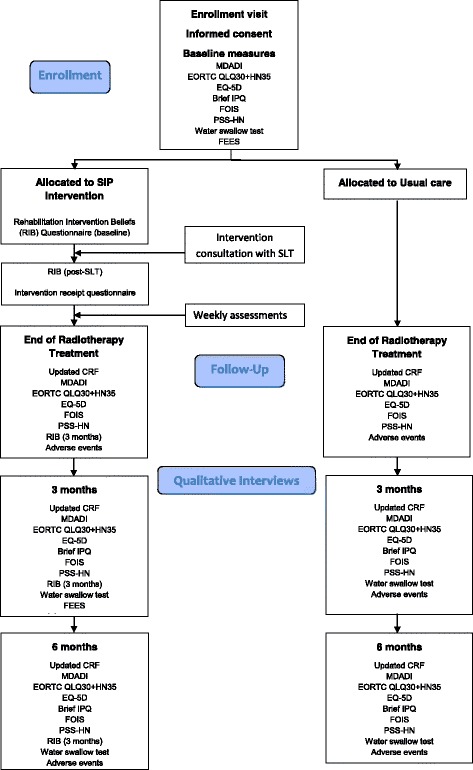
Flow diagram of data collection points for SiP study

A combination of patient-reported outcome measures (PROMs), adherence measures and clinical swallowing assessments will be used prior to intervention (baseline), at the end of treatment, 3 and 6 months post-treatment (see Fig. 1). Data will provide key estimates of the variability for primary and secondary outcomes to inform the best primary outcome measure and sample size estimations for a future trial. Where possible, data are being collected during or after routine clinic appointments in order to enhance retention and complete follow-up.

### Patient-reported outcome measures

Swallowing function will be measured by the MD Anderson Dysphagia Inventory (MDADI) [26] (likely primary outcome measure). Quality of life will be measured by the European Organisation for Research and Treatment of Cancer (EORTC) Quality of Life Questionnaire for cancer patients (QLQ-C30) with the supplementary Head and Neck Module (HN35) [27, 28] and the EuroQol five dimensions questionnaire (EQ-5D) [29].

### Adherence measures

We have developed a ‘Rehabilitation Intervention Beliefs’ (RIB) questionnaire, based on Cooper et al.’s [30] work on exploring patients’ beliefs about cardiac rehab, to assess beliefs that may affect adherence to the SIP, including perceived necessity, concerns, practical barriers and perceived suitability for the intervention.

**Fig. 2:**
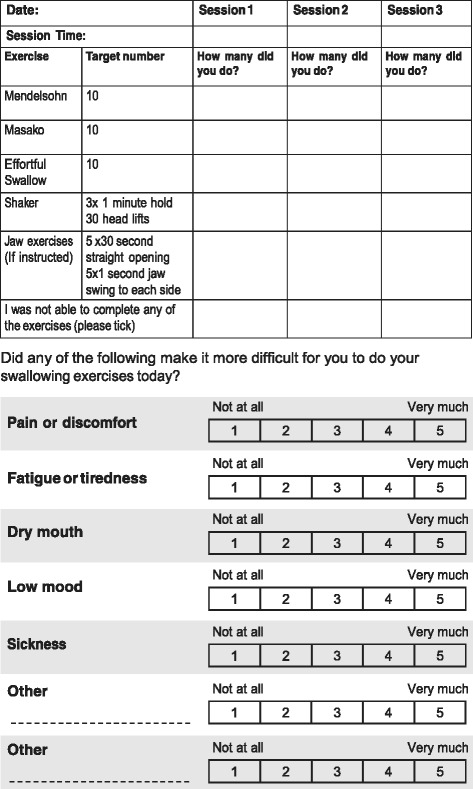
Diary record cards

The RIB and Brief Illness Perceptions (Brief IPQ) [31] questionnaires will assess motivation towards the SIP as well as illness cognitions and emotional representations. At the 3-month data collection point, patients will be asked to complete the post-intervention RIB questionnaire, which includes three additional questions about the patient’s overall experience of the SiP. Structured diary record cards (Fig. 2) will enable recording of daily exercise patterns and frequency, factors which interfered with or promoted adherence, e.g. pain, and any comments about undertaking the exercises. Furthermore, in a small sub-sample of patients, we will pilot the use of technology (e-SIP app) for reporting on adherence; iPads will be provided for the recording of video diaries, enabling real-time data on the performance of swallowing exercises and experiences to be collected.

### Clinical assessments of swallowing ability

1. 100 ml Water Swallow Test (WST) [32]. The WST records the duration and number of swallows required to swallow 100 ml of water. Quick, inexpensive, with good reliability and validity, excellent patient and clinician acceptability, it is sensitive to measure changes over time and follows the expected pattern in differentiating between different cancer treatment regimes. Unlike instrumental tests, the WST does not provide any information on swallow pathophysiology. It has therefore been selected to provide interim information about changes to swallowing function, as an adjunct to the more expensive Fibreoptic Endoscopic Evaluation of Swallowing (FEES) assessment.
2. Performance Status Scale for HNC (PSS-HN) [33]. This three-item scale evaluates dietary texture restrictions and social eating. It has excellent discriminatory properties, good inter-rater reliability and sensitivity to differences in performance and change over time.
3. FEES. This instrumental test allows quantification of endpoints of swallowing pathophysiology, swallowing efficiency and airway invasion (laryngeal penetration and aspiration) [34]. The alternative to FEES—videofluoroscopy (VF) [35]—exposes patients to radiation, is more expensive, less practical to implement and is associated with greater loss to follow up. Blinded FEES assessments will be analysed by the SLT consultants using reliability testing. This test is used routinely in clinical practice but is not carried out for all head and neck cancer patients. An information leaflet is always provided to patients and informed consent is taken before the FEES is carried out.
4. Functional Oral Intake Scale (FOIS) [36]. This scale is scored according to a level that best describes the patient’s oral intake. There are seven levels from level 1: nothing by mouth to level 7: total oral diet with no restrictions.

### Intervention (SiP)

**Fig. 3:**
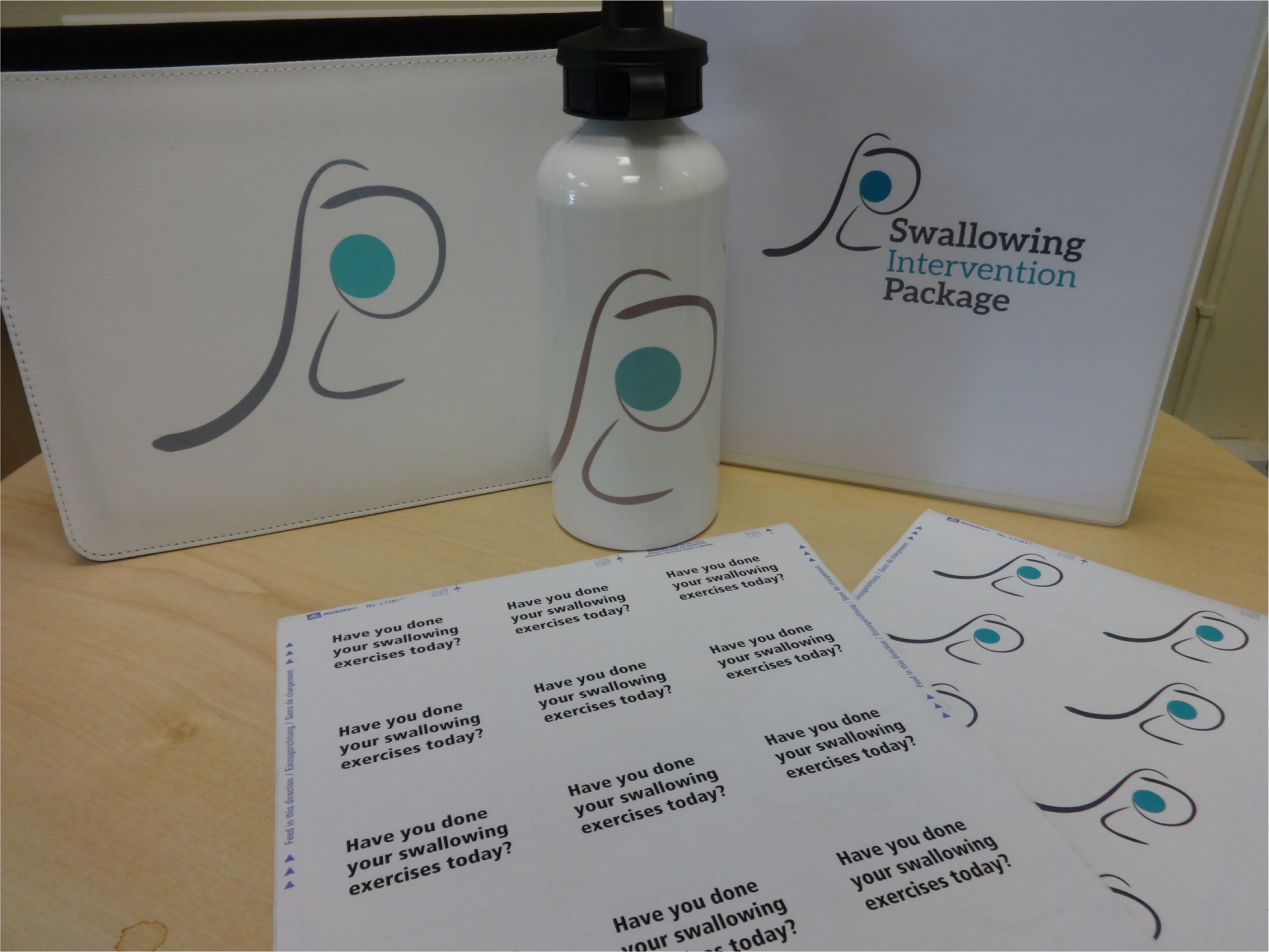
Picture of patient manuals and water bottle

**Fig. 4:**
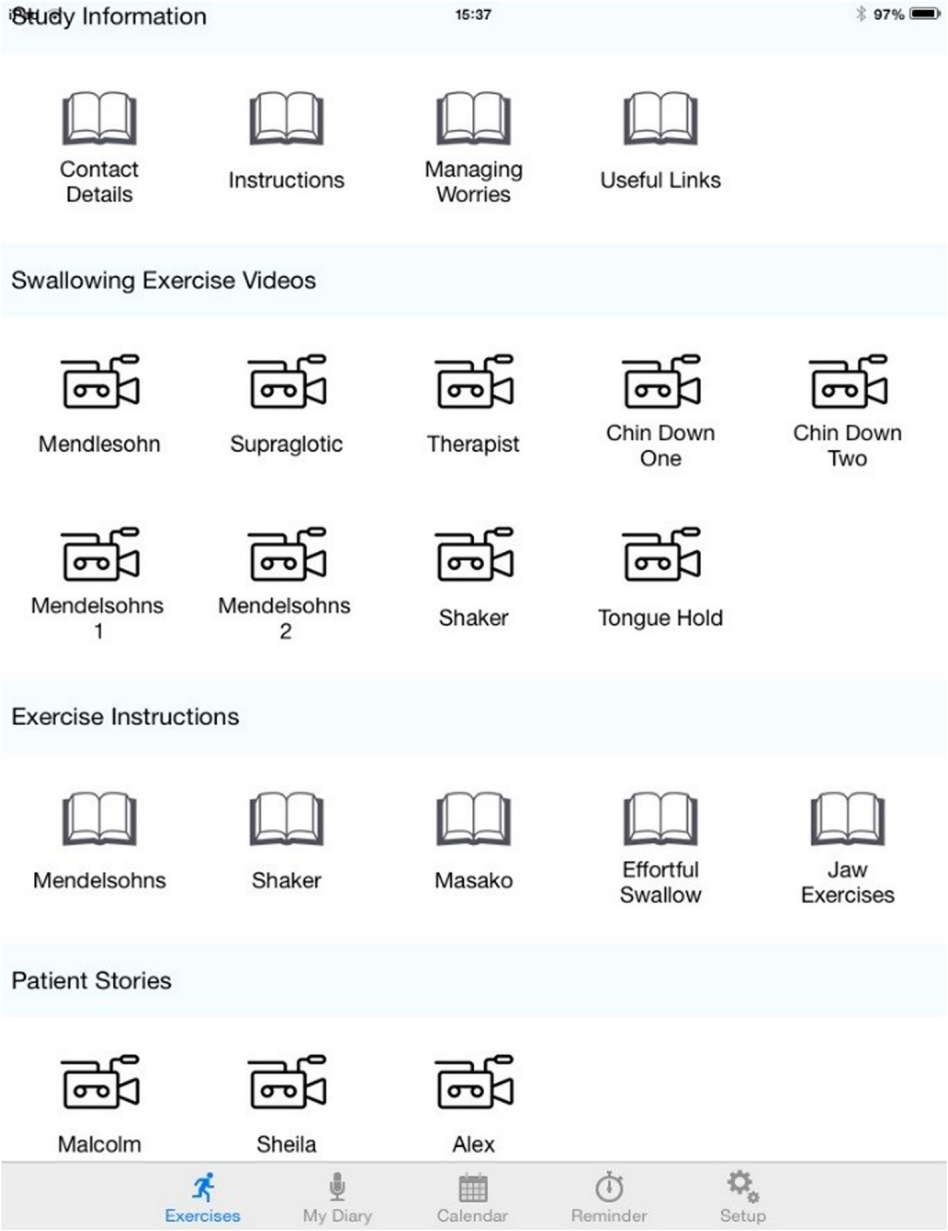
Screenshot of e-SiP

**Fig. 5:**
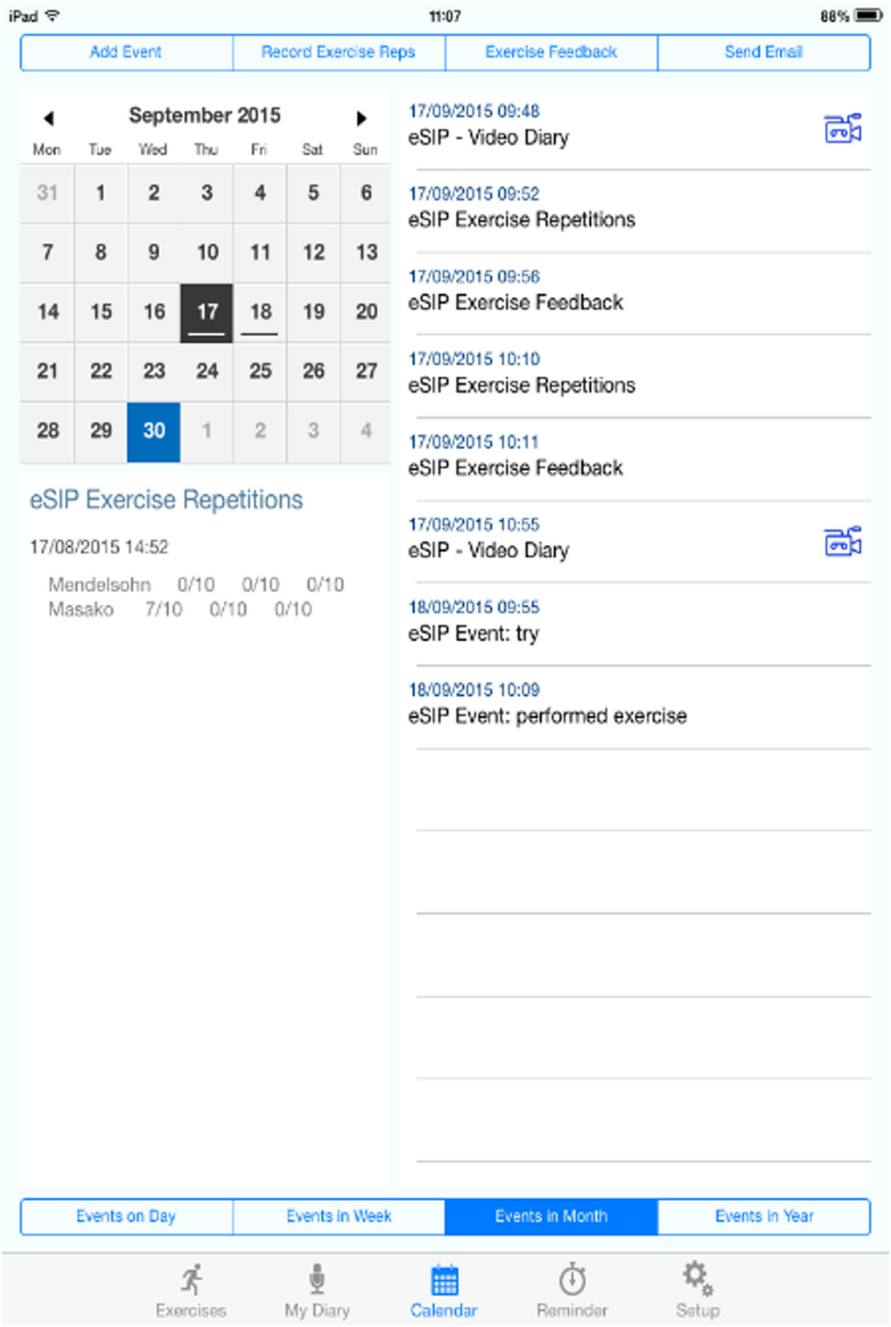
Screenshot of e-SiP

**Fig. 6:**
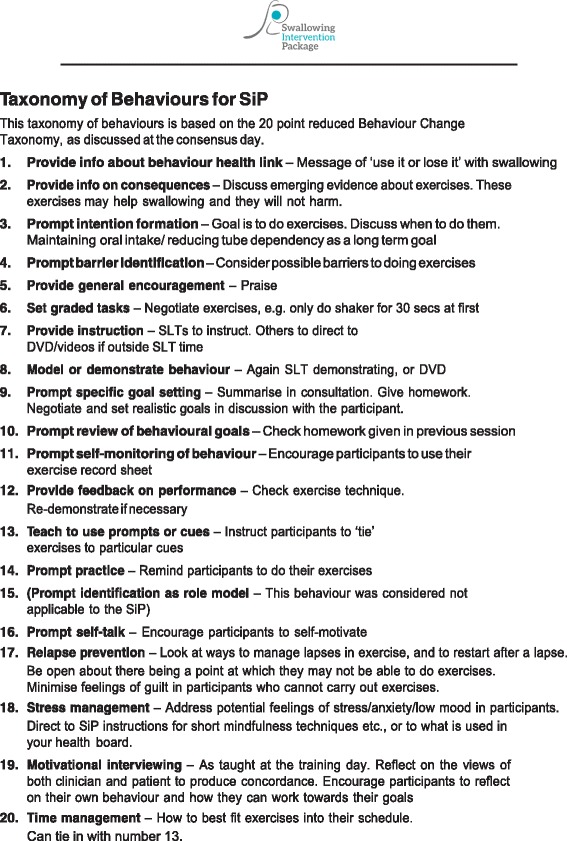
Taxonomy of behaviours for SiP

The SLT based in the relevant centre will teach the exercise programme and provide supporting materials, including diary record sheets, a study water bottle, a SiP patient manual^2^ (Fig. 3), swallowing exercise videos and an iPad containing the e-SiP (which will be piloted in a small number of patients) (Figs. 4 and 5). SLTs reviewed the current literature on prophylactic exercises alongside the evidence base for specific swallowing exercises linked to the pathophysiology of post-irradiated dysphagia and achieved consensus over the exercise regime for SiP (see Table 1). A staff manual was developed based on the Behaviour Change Taxonomy (BCT) [37]. The original 26 point taxonomy was reduced to a 20-point taxonomy using staff consensus. During the consensus workshop conducted as part of phase 1, a multi-disciplinary group of staff reviewed the BCT and devised the manual based on how this could be applied in the SiP intervention (for example, in setting goals with patients), both for initial delivery and ongoing patient support. An example page from this manual is shown in Fig. 6. The aim of the manual is to develop maximum intervention fidelity across the different delivery sites [38]. A small number of interventions will be recorded or observed to check the inclusion of particular behaviours displayed by patients or staff during intervention delivery.

**Table 1:** Swallowing exercises

	Target number for each day		
Exercise	Session 1	Session 2	Session 3
Mendelsohn	10	10	10
Masako	10	10	10
Effortful swallow	10	10	10
Shaker	3× 1-min hold	3× 1-min hold	3× 1-min hold
	30 head lifts	30 head lifts	30 head lifts
Jaw exercises (if instructed, once patient is no longer able to insert three fingers between top and bottom teeth)	5× 30-s straight opening	5× 30-s straight opening	5× 30-s straight opening
	5× 1-s jaw swing to each side	5× 1-s jaw swing to each side	5× 1-s jaw swing to each side

SLTs make the decision whether to reduce/remove an exercise depending on the individual patient

This initial consultation will take place before the start of CRT. At the end of the consultation, the SLT will complete an intervention receipt questionnaire, detailing the main components of the intervention delivered and an assessment of the patient’s understanding and motivation towards the intervention.

### e-SiP delivery

Patients in Tayside who have expressed willingness to trial the e-SiP app version will be provided with an iPad during their initial intervention delivery appointment. They will also be asked to sign an additional consent form which sets out their responsibilities in taking care of the iPad and the boundaries for appropriate use. Two user guides are included with the iPads; the patient user guides details the use of the e-SiP app. In addition, the staff user guide explains the additional log-in functionality that staff may access to change features, e.g. the number of exercise repetitions, within the e-SiP app for that particular patient. All staff delivering the iPad have received training on encouraging the use of the e-SiP, e.g. recording video diaries, over and above the basic staff manual for the SiP delivery.

### On-treatment review

During CRT, either the SLT or clinical nurse specialist (CNS) will review each patient regularly, according to usual on-treatment review practice in each centre. Symptoms will be managed according to local treatment and supportive care protocols. Additional on-treatment review opportunities with nurses, therapy radiographers and dietitians will be used to provide feedback on effort and progress, encourage maintenance of the intervention and monitor adherence to exercises. A weekly assessment log based on the BCT will enable practitioners to document the content of interactions about swallowing that they have had with the patient during treatment, allowing resource use, intervention fidelity and costs to be estimated. In addition, a purposive sample of consultations (pre-, during and post-treatment) between SLTs and patients in each centre will be observed or recorded, using an observation checklist [39]. Patients will be encouraged to bring a family member or friend with them for intervention visits, in order that they can facilitate and support aspects of the SiP at home.

### Data monitoring committee

Although considered, it was deemed that the project did not justify the role of a separate Data Monitoring Committee (DMC). The intervention consists of clinically validated swallowing exercises and assessments that are used already in clinical practice, and there are no anticipated risks to participants over and above those related to the patient’s cancer treatment. The role of the DMC has therefore been subsumed into the function of the Study Steering Committee, who have overseen the monitoring of adverse events. Within this, MW, ND, N H-W and EK, have formed the Data Management Team to discuss day-to-day queries concerning data entry and analysis.

### Qualitative evaluation

Ten to 15 patient participants (and 5 carers) will be invited for an in-depth interview between 6 weeks and 3 months after treatment finishes. Some of these interviews may be joint patient/carer dyads. Purposive sampling will be used to maximise the potential for including participants from different socio-demographic backgrounds, with a range of swallowing difficulties, who adhered to the exercises to varying degrees and used different adherence support, e.g. paper based or e-SiP. Both those who participated in the intervention and a small number from the ‘usual care’ group will be included. The main research fellow for the study will (a) conduct face-to-face or telephone interviews with patient and carer participants, to explore experiences, challenges and adherence to SiP and to identify strategies used by patients (and carers) to overcome any challenges, including which support measures were most and least useful and (b) undertake face-to-face or telephone interviews with 15 key members of staff (SLTs, CNS’, clinic nurses, radiographers, dietitians) from feasibility study sites to elicit views of the SiP and e-SiP, problems with implementation and perceptions of patient outcomes [40]. These data will provide important insights into the intervention refinements necessary for a future RCT.

### Data analysis

All data will be entered from pseudonymised case report forms (CRFs) onto a secure password protected database based at the study coordinating centre. Quantitative data will be entered onto a SPSS v21 database using a coding manual. Qualitative data will be transcribed verbatim and entered into N-VIVO for analysis. Data will be handled, computerised and stored in accordance with the Data Protection Act 1998. All study data will be retained in accordance with Research Governance and local policy.

Qualitative data will be analysed using framework analysis and quantitative outcome data using SPSS v21 or Stata v14. Descriptive statistics will be used to summarise demographic and clinical data, recruitment rates and proportions of those participating, dropping out and providing usable outcome measures. These will be used to populate a CONSORT study flow chart. Missing data problems will be explored to ascertain if missing at random or not. Patient-reported and clinical swallowing outcomes will be estimated between treatment-as-usual and intervention groups as mean or median scores with respective standard deviations or interquartile ranges. Wherever possible, outcome descriptions, summaries and comparisons will be presented together with estimates of precision in accordance with stipulated CONSORT guidelines (www.consort-statement.org). As this is a feasibility study, the data will not be pooled with any future main trial data. This study is not powered to detect significant differences between groups, and therefore, no formal tests of statistical significance will be undertaken.

Statistical models utilising the repeated measures, with baseline measures used as covariates in statistical models will be used to ascertain likely key independent predictors explaining variability of the range of possible outcome measures in this feasibility study. We do not expect to achieve significant differences from baseline to follow-up measure(s) between or within groups as this study is inadequately powered and is quasi-experimental; however, we expect to estimate relative effect sizes (and 95 % CI) for each potential main outcome measure, correlation between measurements, and intra-cluster (site) correlation coefficient for use in sample size simulations for a future randomised trial; this study aims to ascertain if the data collection is sufficient to yield usable data in hypothesis testing in a definitive multi-centre trial and to assess whether the direction of effect is in favour of the intervention. Preliminary psychometric validation of the RIB will also be undertaken, including Cronbach’s alpha (for internal consistency), correlation analyses for test-retest reliability and construct validity. The EQ-5D will be used to calculate quality-adjusted life years (QALY) gains and used along with an assessment of resource implications to inform a full economic evaluation in a future definitive trial. With respect to the analysis of the e-SiP, the e-SiP itself is not considered part of the intervention (it does not represent any change in actual exercise protocol), but is a means of testing different ways of data collection, improving any missing data problem and supporting adherence. Although we intend to incorporate the e-SiP data into our analysis, our sample of patients using the e-SiP will be very small due to the available number of iPads. However, we will conduct a sensitivity analysis whereby we analyse the data with and without the e-SiP sub-sample to ascertain if there are any changes to effect size and variance. We will assess e-SiP use to see which aspects of the app patients use most and to conduct a comparison of entries between the paper and e-SiP diaries.

## Discussion

The potential for swallowing exercises to improve short- and long-term outcomes in patients with head and neck cancer is increasingly recognised, but the evidence base remains limited.

The support of specialist SLTs and other members of the MDT is known to be important, but the extent to which patients routinely have access to SLTs before and during treatment is extremely variable [41]. This is due in part to our limited knowledge about the best ways to deliver swallowing interventions and support people who have or are at risk of developing swallowing difficulties as a result of treatment for HNC.

Patients undergoing CRT experience significant fatigue and other side effects, which are likely to challenge their ability to perform swallowing exercises, but the experiences of patients and the factors influencing adherence to exercises on a day-to-day basis are largely unknown. In this study, data collected relating to patient and carer attitudes, beliefs and experiences during and after CRT will provide important insights into potential barriers and facilitators to exercise adherence in a range of real-life contexts. These will inform future studies.

This collaborative study has taken a unique approach to the development of a patient-centred and evidence-based swallowing intervention, which aims to encourage and support adherence to daily swallowing exercises during and after CRT, whilst being practical for SLTs and MDTs to implement for a wide range of patients. The introduction of the e-SiP provides an exploration of the use of technology in delivering this intervention, which might be especially pertinent given the increasing numbers of younger HNC patients.

We have worked in partnership with SLTs, patients and carers to develop this complex intervention. The study provides an opportunity to examine the feasibility of delivering and participating in a supported swallowing intervention across several different NHS sites and will provide the evidence needed to refine intervention and study processes for future research. We will collect a broad range of qualitative and quantitative data, including outcome measures, clinical assessments and interview. This will result in a large amount of diverse data which will inform a definitive multi-centre trial, based on sound theoretical and empirical foundations, feasible for patients and staff and implementable on a wide scale.

### Dissemination plans

We will continue to engage and share findings with a wide range of HNC specialists and SLTs across the UK and Europe. Our study is on the National Cancer Research Institute (NCRI) portfolios for Psychosocial Oncology and Survivorship and Head and Neck Cancers. We will discuss our findings and their implications with the NCRI Clinical Studies Groups, Macmillan and HNC charities and with staff and patients from participating NHS Boards. We will present our work at National and International meetings and will submit findings for publication in clinical peer-reviewed journals.

## Trial status

The proposal for this study was peer reviewed and then funded by the Chief Scientist Office in July 2014 (ref: CZH/4/1052). Progress reports are submitted to the funder on a regular basis, but no further input to the design, analysis, interpretation or writing up of the data is given by the funder. iPads have been loaned by Throat Cancer Foundation. Phase 1 was approved by NRES Committee North East—Newcastle and North Tyneside 2 in October 2014 (REC:14/NE/1168; NRS R&D: NRS14/ON593). Phase 2 was approved by the East of Scotland Research Ethics Service (EoSRES) in October 2015 (REC: 15/ES/0106; NRS R&D: NRS15/ON703), and participants are being recruited at time of submission. All protocol amendments are communicated to relevant R&D, ethics and research teams. Sponsorship, indemnity and insurance are provided by the University of Stirling. Target completion date for phase 2 is February 2017. We are happy to share the protocol and supporting documents with other researchers.

## Abbreviations

CNS, clinical nurse specialist; CRF, case report form; CRT, chemoradiotherapy; EORTC HN35, European Organisation for Research and Treatment of Cancer Quality of Life Questionnaire Head and Neck; EORTC QLQ30, European Organisation for Research and Treatment of Cancer Quality of Life Questionnaire 30; FEES, Fibreoptic Endoscopic Evaluation of Swallowing; FOIS, Functional Oral Intake Scale; HNC, head and neck cancer; IMRT, intensity-modulated radiation therapy; IPQ, Illness Perceptions Questionnaire; MDADI, MD Anderson Dysphagia Inventory; NCRI, National Cancer Research Institute; PROMs, patient-reported outcome measures; PSS-HN, Performance Status Scale for Head and Neck Cancer Patients; RIB, Rehabilitation Intervention Beliefs; RT, radiotherapy; SiP, Swallowing intervention Package; SLT, speech and language therapist; VF, videofluoroscopy; WST, Water Swallow Test

## Footnotes

1 For the purposes of this protocol ‘CRT’ includes patients undergoing chemoradiotherapy or radiotherapy, either as primary treatment or after surgery.

2 This includes information and advice on normal swallowing, importance of maintaining swallow, tips for eating and drinking, swallowing exercises, diary record cards, strategies for managing distress and anxiety and resources for further information and support
